# Geometric Theory Predicts Bifurcations in Minimal Wiring Cost Trees in Biology Are Flat

**DOI:** 10.1371/journal.pcbi.1002474

**Published:** 2012-04-12

**Authors:** Yihwa Kim, Robert Sinclair, Nol Chindapol, Jaap A. Kaandorp, Erik De Schutter

**Affiliations:** 1 Computational Neuroscience Unit, Okinawa Institute of Science and Technology, Okinawa, Japan; 2 Mathematical Biology Unit, Okinawa Institute of Science and Technology, Okinawa, Japan; 3 Section Computational Science, Faculty of Science, University of Amsterdam, Amsterdam, The Netherlands; Indiana University, United States of America

## Abstract

The complex three-dimensional shapes of tree-like structures in biology are constrained by optimization principles, but the actual costs being minimized can be difficult to discern. We show that despite quite variable morphologies and functions, bifurcations in the scleractinian coral *Madracis* and in many different mammalian neuron types tend to be planar. We prove that in fact bifurcations embedded in a spatial tree that minimizes wiring cost should lie on planes. This biologically motivated generalization of the classical mathematical theory of Euclidean Steiner trees is compatible with many different assumptions about the type of cost function. Since the geometric proof does not require any correlation between consecutive planes, we predict that, in an environment without directional biases, consecutive planes would be oriented independently of each other. We confirm this is true for many branching corals and neuron types. We conclude that planar bifurcations are characteristic of wiring cost optimization in any type of biological spatial tree structure.

## Author Summary

Morphology is constrained by function and vice-versa. Often, intricate morphology can be explained by optimization of a cost. However, in biology, the exact form of the cost function is seldom clear. Previously, for many different natural trees authors have reported that most bifurcations are planar and we confirm this here for branching corals and mammalian neurons. In a three-dimensional space, where bifurcations can have many shapes, it is not clear why they are mostly planar. We show, using a geometric proof, that bifurcations that are part of an optimal wiring cost tree should be planar. We demonstrate this by proving that a bifurcation that is not planar cannot be part of an optimal wiring cost tree, using a very general form of wiring cost which applies even when the exact form of the cost function is not known. We conclude that nature selects for developmental mechanisms which produce planar bifurcations.

## Introduction

Bifurcations are observed widely in nature [1], such as in dendrites and axons of neurons [2], plants [3], rivers, capillaries [4], [5], [6], bronchi and tracheal systems [7], [8], [9] and octo- and scleractinian corals [10], [11]. Most studies have focused on characterizing tree geometry [7], [12], [13], [14], [15], [16] with an emphasis on the implications for function [17], [18], [19] or in relation to optimization processes [4], [8], [20], [21].

Overall, much less attention has been given to bifurcation properties. While it has occasionally been reported that bifurcations tend to be planar in different natural trees such as in neurons [22], in arterial systems [6], in lungs [9] and in plants [3], this property has not been systematically studied or properly explained [3].

In this paper we first characterize planarity in two very different types of spatial tree: the branches of corals and the dendrites of neurons. The point of doing so is to demonstrate evolutionary convergence, a feature one expects to be exhibited by any true optimization principle in biology [17]. We next investigate whether we can use the theory of Steiner trees to explain this phenomenon. Steiner tree theory is an active research field [23] that studies wiring cost minimization [24], mostly in two dimensions. It has been used as a framework for understanding wiring cost optimization in neurons [25], [26] and other naturally occurring trees [27]. Steiner trees minimize edge costs by allowing the addition of extra nodes (Steiner points) whenever these reduce the total wiring cost. When the costs are defined as the Euclidean distances between nodes, these are Euclidean Steiner trees [28]. Minimal Euclidean Steiner tree bifurcations in space must be planar [29], but they also require 120° angles between adjacent edges [28], [29].

We show that the bifurcations we studied are not compatible with the Steiner tree paradigm and instead propose a new general theoretical framework that has the advantage of not requiring any specific assumptions about wiring cost beyond an increase with branch length. This theory provides for the first time an explanation for flat bifurcations that can be applied to all kinds of natural trees.

## Results

### In the coral *Madracis* and neuronal dendrites, bifurcations are mostly planar

**Figure 1:**
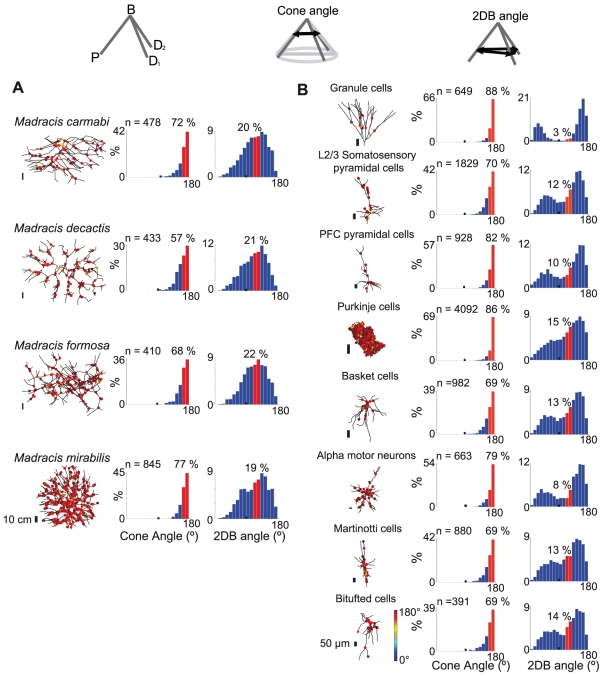
Geometric measurements of the scleractinian coral *Madracis* and neuronal dendritic bifurcations show that bifurcations are mostly planar, but bifurcation angles are not always Steiner tree optimal. Measurements were done on bifurcations composed of a parent (P) and two daughter branches (D_1_, D_2_). For each case an example of the very different coral and dendritic tree morphologies is shown (left), the distribution of cone angles [22] as a quantification of the shape of a bifurcation (center) and the distribution of the angles between the branches (right). **A.** Samples of coral bifurcations from 4 coral species. **B.** Samples of dendritic bifurcations of 8 neuron types. The cone angle values are marked in color in the range of 0 to 180° on the samples themselves (see scale at the bottom right). The histogram of cone angle distributions peaks at 180°, showing a marked tendency towards planarity. The bifurcation angle (2DB) distribution for both corals and neurons shows that only a small proportion of angles are close to 120°.

We examined whether planarity is a common property of natural structures by quantifying flatness using cone angles [22]. The cone angle of a bifurcation is the aperture or opening angle of a right circular cone which contains the three bifurcation branches within its surface and has its apex at the branching point. A flat bifurcation has a cone angle of 180°. We examined the properties of bifurcations in two very different biological data sets. The first data are corals where planarity has not been demonstrated previously: we measured cone angles in digital reconstructions of four species of the branching coral *Madracis* (Figure 1A, Table S1). Secondly, we extended the previous observation of flat bifurcations in visual cortex pyramidal neurons [22] to eight different mammalian neuron types (Figure 1B, Table S1). We found that despite their rich and varied spatial morphologies, in all coral species (Figure 1A) and all neuron types (Figure 1B) most dendritic bifurcations (57 to 88%) were close to planar (between 160° and 180°), confirming that planarity is a general property of coral and of neuronal dendritic trees and axons (Figure S1A).

### Neuronal dendritic and coral bifurcation geometries are not of Steiner type

We next asked whether flat bifurcations could be explained by considering these trees to be Steiner trees. We tested whether coral or neuronal dendritic bifurcation angles between any of the 3 branches in a bifurcation tend to be close to 120°, as must be the case for minimal Euclidean Steiner trees. Although the peaks of branching angle distributions of corals were near 120°, only 19 to 22% of branches had branching angles close to 120° (between 110° and 130°) (Figure 1A). There were differences between coral species: while most had a single peak in the distribution, *Madracis mirabilis* had two separated peaks. Conversely, in neurons all branching angle distributions were bimodal with peaks smaller and larger than 120° (Figure 1B). Consequently only 3 to 15% of angles were close to 120°, as was also shown in other studies [25], [26].

The fact that neurites and coral skeletons are not of Euclidean Steiner tree type leaves unanswered the question of why their bifurcations are mostly planar. Can this be explained by another optimality-based principle?

### Random bifurcations

**Figure 2:**
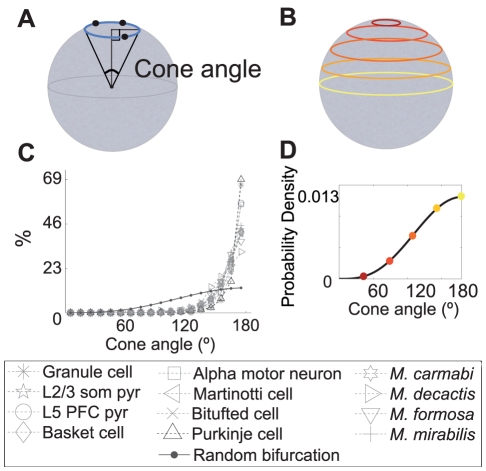
Random bifurcations have a tendency to be planar. **A.** Diagram showing how random bifurcations can be mapped onto a unit sphere. The bifurcation point is fixed at the center of the sphere and non-bifurcation points are projected onto its surface. Intuitively, the probability of finding a cone with cone angle 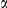 can be thought of in terms of choosing three non-bifurcating points that fall onto the base circle of this cone, i.e. the intersection of the cone with the sphere. **B.** The circumference of the base circle gets larger as the cone angle increases. **C.** Overlay of the random bifurcations' distribution of cone angles with the biological distributions shows that the distribution of all biological bifurcations deviates significantly from the random one (KS test, p-value = 10^−5^). **D.** The probability distribution for random bifurcations is 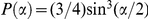 (where 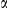 is in radians). The probability of cone angles >160° is 26%.

We first checked the null hypothesis that random bifurcations are not planar. To investigate this, we calculated the probability distribution of cone angles for bifurcations with a random orientation of the 3 branches (Figure 2). For this calculation, the bifurcation point was located at the center of a unit sphere and all other points were located on the sphere surface, but the results apply also to 4 random points in space defining a bifurcation (Text S1). For a bifurcation to be on a cone with cone angle 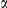, all non-bifurcation points need to lie on the circular intersection, of the cone with the unit sphere, whose circumference is 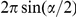 (Figure 2B). Even taking singular cases into account, the probability distribution for cone angle 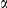 is in fact (Text S1) given by
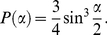
(1)Since the circumference of the base circle increases with increasing cone angle, the probability distribution of 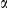 also increases, with a maximum at 180° (Figure 2D). Though the finding that even random bifurcations have a tendency to be flat may be surprising, we found that in corals and neurons the proportion of close to planar bifurcations was always much more pronounced (Figure 2C, p = 10^−5^).

For the neural data we also excluded histological artifacts as a cause of planarity (Text S1 and Table S2).

### A property of wiring cost optimal trees

Having excluded trivial explanations for the planarity of bifurcations, we returned to the issue of wiring cost minimization and discovered that indeed it is possible to prove that an optimal wiring cost tree, even with varying wiring cost, should have planar bifurcations. We assumed that a number of regions (which may be simply points) containing terminal or target points are given, which are connected by a wiring cost minimizing tree. No causal or teleological relationship between these regions and the growth of the tree is implied. Our proof is essentially a test of optimality of any given spatial tree. Given a tree, which must necessarily have terminal points, we asked whether it could be optimal with respect to those points. Whether or not the terminal regions or points were specified before or during growth, or were simply the terminal points of the tree at the moment we observed it, does not enter in to our mathematical proof. The wiring cost we considered is a sum of individual edge costs, each of which should be continuous and strictly increasing with edge length. This type of wiring cost is general enough to describe not only a total wiring cost in the usual sense, but also the total cost of paths from each terminal point or intermediary target point to a root, or any linear combination of these costs [30], [31], [32]. Moreover, this type of wiring cost can also take into account conflicting cost functions previously proposed in neuroscience such as volume, surface and neural conduction time [26], [33], since all of them increase with wiring length, as well as cost functions that might vary during development or are specific to each branch [34]. See Text S1 for details.

**Figure 3:**
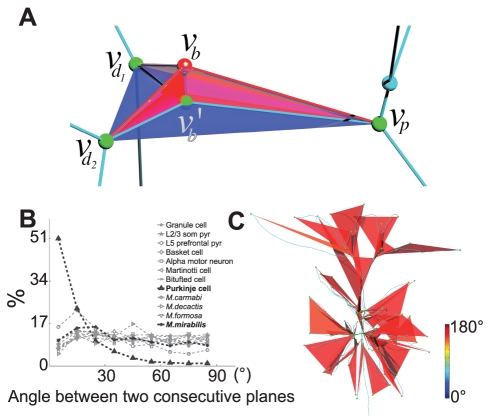
A bifurcation in an optimal wiring cost tree has to lie on a plane and consecutive planes are independent of each other. **A.** Projecting the red bifurcation point (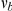) onto the blue bifurcation plane reduces the costs of all three edges involved, and therefore the total cost of the entire tree. Note that in this example the bifurcation plane is defined by neighboring bifurcation points (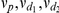), but some or all of these could be replaced by fixed or terminal points. **B.** Most of the distributions of angles between consecutive bifurcation planes approximate the uniform distribution, except for Purkinje cells, L5 PFC pyramidal cells and *M. mirabilis*. Data from the other 6 neuron types and 3 coral species were statistically consistent with a uniform distribution (p>0.05, one-sample Kolmogorov-Smirnov test, considering only basal dendrites for L2/3 pyramidal neurons). **C.** Example of bifurcation planes in a L2/3 somatosensory cortex pyramidal cell. The bifurcation planes are color coded according to their cone angles.

Rather than constructing an optimal solution, which is known to be an NP-hard problem [35], we investigated properties any optimal tree must possess. As in the Steiner tree problem, we allowed additional points to be added. We began by considering an arbitrary bifurcation point (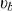) and the three points it connects to (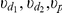) (Figure 3A). These three points define a bifurcation plane. If the bifurcation point is not in the bifurcation plane (i.e. if the bifurcation is not planar) we asked whether it can be part of an optimal tree. We showed that one can always move this bifurcation point, unless explicitly forbidden by an imposed biological necessity or obstruction, onto the bifurcation plane in such a way (normal projection) that the three edges of the bifurcation are all shortened. The fact that three edges of the tree can be shortened, without making any change to the other edges of the tree, means that the entire tree containing the non-planar bifurcation cannot have been optimal. Thus, we could conclude that all bifurcations in any optimal tree must be planar.

What does the shape of optimal bifurcations tell us about the shape of the whole tree? The shape of a tree is determined by its target points, and when these points do not provide directional biases our result suggests that there is no need for any correlation between the orientations of the bifurcation planes. To further test this idea, we measured the angles between consecutive bifurcation planes in the neuronal and coral data (Figure 3B). These experimental distributions clearly approximate a uniform distribution of angles from 0 to 90° for all, except for Purkinje neurons and the skeletons of *Madracis mirabilis* colonies. *Madracis mirabilis* is known to be geometrically exceptional within the genus *Madracis* as a result of its unusually regular branching patterns [16] which are strongly suggestive of developmental constraints overriding purely geometrical optimality considerations. In the case of Purkinje cells, the symmetry breaking induced by the parallel fibers allows only a globally planar solution to be optimal, meaning that the dendritic tree is flat and all the bifurcations lie on the same plane [32], just as the symmetry breaking induced by water currents can result in globally planar fan coral morphologies [36] in octocorals. Similarly developmental controls during lung growth cause the bifurcations which are planar to occur in either the same plane or in an orthogonal plane to the preceding one [9]. These apparent exceptions are important because they strengthen our case for evolutionary convergence between neuronal and octo- and scleractinian coral skeleton morphologies.

## Discussion

We have confirmed that diverse natural trees have a large proportion of flat bifurcations and proposed a general theory that shows that this must be expected for optimal wiring cost trees. Unlike Euclidean Steiner tree theory, in which the cost of an edge is nothing other than its length, our general theory allows each individual edge to have its own cost defined in terms of any continuous, strictly increasing function of length. The fact that we do not need to specify the actual functions any further is a strength of our approach. It is not whether edges make 120° angles which is characteristic of optimal wiring cost [26], but rather the fact that bifurcations are always planar. Our theory extends to bifurcations of the axons of neurons (Figure S1), where planarity has previously been overlooked [26], and planar bifurcations observed in arteries [4], [5], [6], mammalian lungs and invertebrate trachea [9], [37], plants [3], or even nanotubes [38].

Our theory does not suggest specific mechanisms for achieving planar bifurcations and different organisms will choose different strategies. In the case of neurons, dendritic planarity has been attributed to tension forces flattening the dendrite during development [39]. However tension may not suffice to explain dendritic planarity in complex extracellular space. Instead, neuronal dendritic planar bifurcations could arise during growth via mechanisms such as pruning [40], [41], [42] where branches with high wiring cost are eliminated, or with simple growth rules [43] like, repulsion between branches [44], [45], or via genetic control [9], [37], [46].

The rich literature on the optimization of neuronal trees has mostly focused on larger scale tree morphology. Because our theory defines wiring cost optimization in a very general way, it is independent of any particular biophysical model of neuronal tree growth, restructuring or maintenance [39], [40], [47], [48]. Our results are also consistent with different explanations for the appropriate definition of cost, be it minimal energy expenditure of processes [22], flow across a bifurcation [8], [39] involving functional considerations such as connectivity [49], or other models which have been proposed [26], [43], [50].

Conversely, much less attention has been given to the optimization principles underlying tree-structures in coral biology. By some authors it has been hypothesized that the coral is optimizing branch spacing and compactness to maximize the internal flow velocity between the branches of a colony that sustains the mass transfer rate into and out of the colony [51]. There is a strong morphological plasticity in corals due to environmental influences [52]. The wiring cost optimization we observed suggests that the branching structure is formed with a minimum amount of material, whether this structure is also optimizing mass transfer in the colony is still unknown. The branching angles distributions for the 4 related species are clearly species-specific. Usually the classical taxonomy (e.g. [53], [54] for *Madracis*) in corals is based on corallite morphologies, while the overall colony is described in a rather qualitative and informal way.

Here, we have provided a general, geometric explanation for the planarity of bifurcations. To the best of our knowledge, it is the first proof of the requirement of planarity of bifurcations in a general spatial framework of wiring cost optimization with varying wiring cost. One of the strengths of our contribution is to show that planarity can be understood at a fairly abstract level and a wide variety of branched tree structures in all areas of biology.

## Materials and Methods

### Data Analysis

#### Scleractinian coral data

We used 3D morphological data of 4 species of the branching coral of the genus *Madracis*. The species analyzed were *Madracis carmabi*, *Madracis decactis*, *Madracis formosa* and *Madracis mirabilis* (See Table S1 for further details). Details of the data acquisition method can be found in [16] .

#### Neuronal Data

We used 3D morphological data of 8 extensively studied mammalian neuron types taken from normal, untreated animals. The morphological data were downloaded from the Neuromorpho.org database [55]. The cells were hippocampal granule cells, Layer 2/3 somatosensory cortex pyramidal cells, Layer 5 prefrontal cortex pyramidal cells, cortical basket, Martinotti and bitufted cells, cerebellar Purkinje cells and alpha motor neurons (See Table S1 for further details).

#### Measurements

The points used for constructing individual bifurcations were either bifurcation points or terminal points (see Text S1 and Figure S2 for an alternative approach). We measured the cone angle of each bifurcation. From a single bifurcation, a cone was constructed using the bifurcation point as the tip and letting the three non-bifurcating points define the surface of the cone. A novel, more robust method to calculate the cone angle is detailed in the Text S1. For the measurement of the angle between two consecutive bifurcation planes, planes were constructed using parent and both daughter points. Two planes were considered consecutive when one of the daughter points of one bifurcation was the bifurcation point of the next bifurcation. A normal vector was constructed from each plane, and the angle between two normal vectors was measured. Since the angles were symmetric around 90°, all the angles that were larger than 90° were reduced to the range of 0° to 90°.

#### Proof of planar bifurcations for wiring cost optimization tree

We prove, under quite general conditions, that bifurcations in a tree optimizing wiring cost must be planar. We do not attempt to provide any method or algorithm for constructing such an optimal tree. Rather, we derive certain properties a globally optimal tree must possess. We work within the framework of geometric graph theory, making use of undirected graphs embedded in three-dimensional Euclidean space (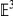). We assume that all trees contain a finite number of points and that all edges are line segments. We consider only trees, since total wiring cost can always be reduced by eliminating one edge in a loop, an operation which does not change connectivity. We also consider only connected trees, since our intended application is to individual dendritic trees. Our proof does not require the existence of any quantity which might be called a force. Instead, we work exclusively with a definition of cost. Our results therefore also apply to systems for which mechanistic explanations of bifurcation planarity do not apply.

Let us imagine a tree involving 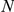 distinct internal vertices 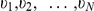 and 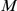 distinct terminal vertices 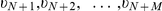 in three-dimensional Euclidean space (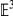). Each vertex is restricted to lie within its own given region (a finite union of closed subsets of 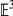 with continuous boundary), which we denote 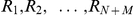, respectively. Internal vertices will not typically be spatially restricted (i.e. their given regions will be 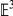), but can be, to accommodate any target points which are not terminal points.

Furthermore, let us suppose that the tree is optimal with respect to wiring cost, in the sense that the positions of the 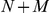 vertices minimize the wiring cost
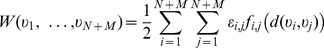
(2)where the elements 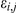 of the adjacency matrix 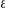 are either (i) zero or (ii) equal to unity if and only if the vertices 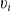 and 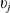 are connected by an edge. 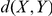 is the Euclidean distance between points 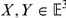. We furthermore assume that the functions 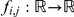 are continuous, strictly increasing and symmetric with respect to their indices, so that 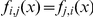 for all 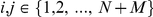 and all 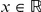. We will make particular use of the fact that they are strictly increasing, and thus have the property that
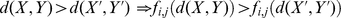
for all 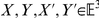. Note that we do not require that the 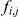 be differentiable everywhere, nor that their first derivative be always positive when defined [56], but we assume continuity because it guarantees the existence of optima [57].

Thus, we are interested in properties of global optima with respect to the optimization problem
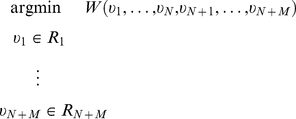
(3)The optimization problem **[3]** does not explicitly touch upon the issue of optimization of tree topology (encoded in the adjacency matrix 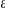). This does not restrict the applicability of our approach, since the properties we establish are independent of topology.

For our purposes, we define a *bifurcation* to be the union of the edges joining an internal vertex 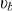 (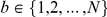) of degree three to its neighbours 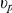, 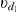 and 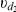, any of which may be a terminal vertex (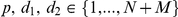), assuming that all four points are distinct. We define a *bifurcation plane* to be the plane defined by the three vertices 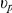, 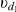 and 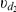 of the bifurcation.

We define a *deformable bifurcation* to be a bifurcation which could be made planar by projecting 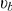 onto the bifurcation plane (Figure 3A). In other words, the given region for 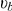 (i.e. 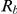) must contain the projection of 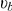 onto the bifurcation plane. By “deformable”, we intend to mean something like “can be flattened”.

Now we will prove that all deformable bifurcations in an optimal tree are planar, by showing that any non-planar deformable bifurcation in a tree implies that the tree cannot be optimal.

Let us assume that 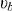 is an internal vertex of degree three in an optimal tree, and that it does not lie in the plane 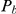 passing through the three points 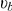 is joined to, which we will identify as 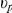, 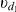 and 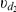, as in Figure 3A. 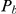 is, by construction, the bifurcation plane. In other words, we assume that this bifurcation is not planar. Let 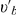 be the unique point in 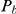 closest to 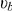. The line passing through 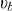 and 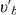 must make a right angle with 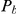.

Since the line segment joining 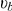 and 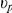 is the hypotenuse of the right triangle 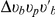, we have 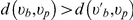 and therefore 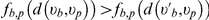.

One can readily verify that the same is true in the cases of the right triangles 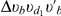 and 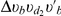. Since 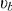 enters into the wiring cost only via the six terms 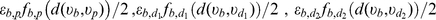, and 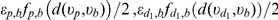 and 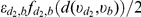, and their sum is 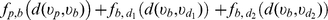 it follows that 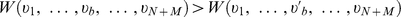.

Therefore, the tree cannot have been optimal, which is a contradiction.

Since our choice of deformable bifurcation was arbitrary, an optimal tree may not contain a non-planar deformable bifurcation. Our proof is complete.

## Supporting Information

Figure S1 Measurements for axonal bifurcations for layer 2/3 pyramidal neurons, basket cells, Martinotti cells and bitufted cells. This axonal data was available for the same neurons for which the dendritic bifurcations are analysed in Figure 1B. The cone angle values are marked in color with the range of 0 to 180°. **A**. Cone angle distributions of the axonal bifurcations **B**. 2D branching angles C. Angles between consecutive bifurcation planes of axons approximate the uniform distribution for Layer 2/3 somatosensory pyramidal cell and bitufted cell (p>0.05, one-sample Kolmogorov-Smirnov test).
(EPS)

Figure S2 **A**. Cone angle distributions for cone angles computed among the points closest to the bifurcation point for each branch in the reconstruction. **B**. Comparison with cone angle distribution for random bifurcations.
(EPS)

Figure S3Definition of the cone angle 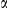, given the four points A, B, C and D of a bifurcation in three dimensions, where A is the point of bifurcation. 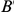, 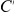 and 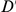 are chosen such that the lengths 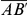, 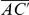 and 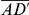 are all equal to unity. 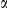 is the angle indicated in the white triangle. The side of the white triangle opposing A is a diameter of the circle passing through 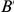, 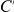 and 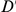.(TIFF)

Figure S4Definition of the distance 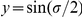. Note that 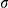 is the angle DAB, 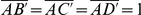, so 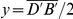.(TIFF)

Table S1 Details of the experimental data used in the paper. Details of coral data can be found in [16]. Reconstructed neurons were acquired from the database Neuromorpho.org [55].
(DOC)

Table S2 Comparisons between bifurcations with different orientations with respect to Z-plane. First two columns: % of bifurcations that belonged to each group. Third column: Jensen-Shannon divergence between the cone angle distributions for the two experimental groups (* two distributions are not different from each other, Kolmogorov-Smirnov test p-value = 0.01). Last two columns: Jensen-Shannon divergence of experimental groups with the cone angle distribution for random bifurcations.
(DOC)

Text S1 This file contains computing cone angle from the first bifurcation segment, assessing the possible effect of shrinkage artefacts in neuronal reconstruction data, extended comments relating to the proof, numerical evaluation of the cone angle and cone angle distribution for random points.
(PDF)
