# Supplementary figures and images for: Geometric Theory Predicts Bifurcations in Minimal Wiring Cost Trees in Biology Are Flat
Source: PLoS Comput Biol. 2012 Apr 12;8(4):e1002474. doi: 10.1371/journal.pcbi.1002474 (PMC3325189; doi:10.1371/journal.pcbi.1002474)

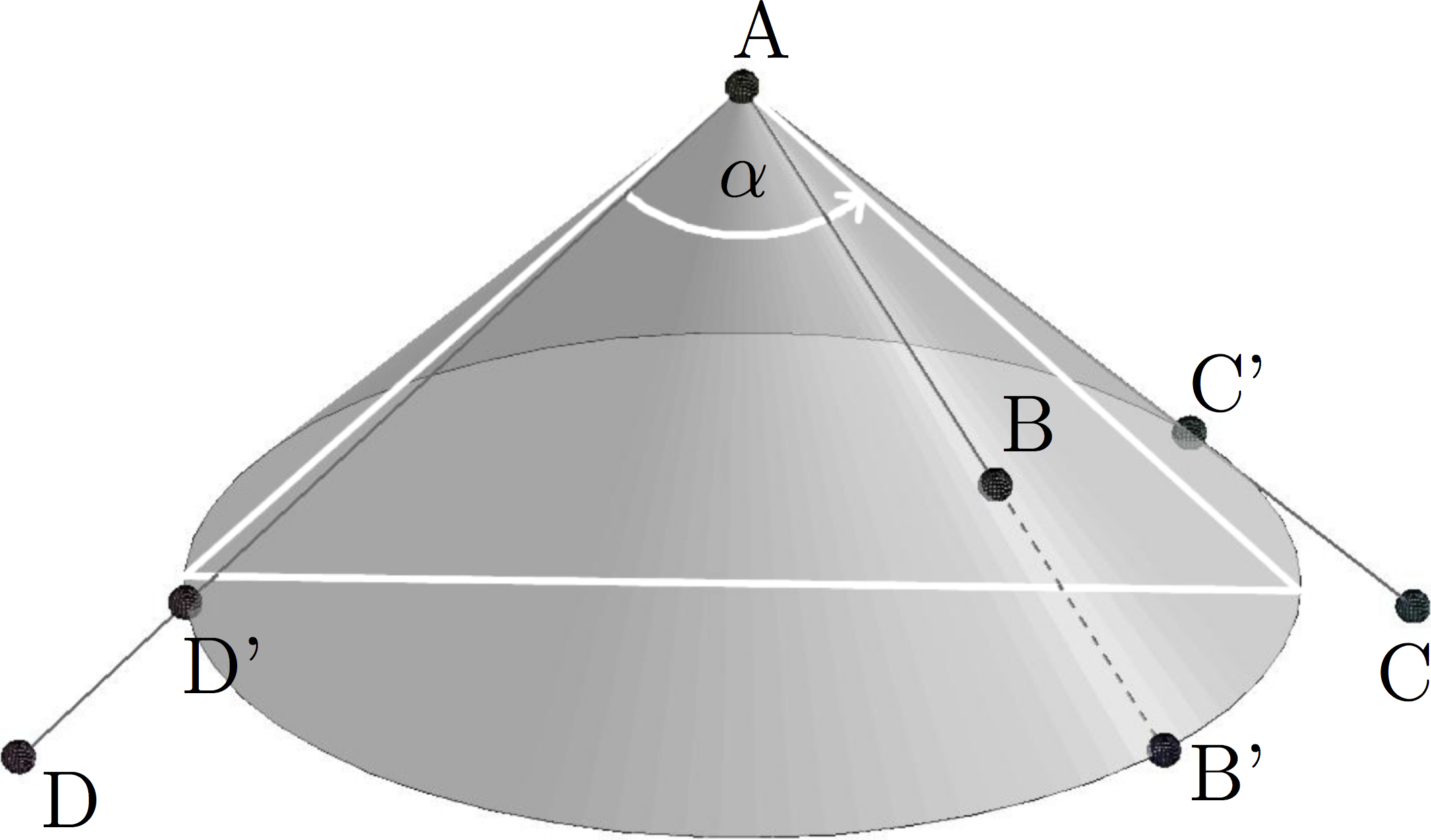

Supplement: Figure S3 — Definition of the cone angle , given the four points A, B, C and D of a bifurcation in three dimensions, where A is the point of bifurcation. , and are chosen such that the lengths , and are all equal to unity. is the angle indicated in the white triangle. The side of the white triangle opposing A is a diameter of the circle passing through , and . (TIFF) [file pcbi.1002474.s003.tif]

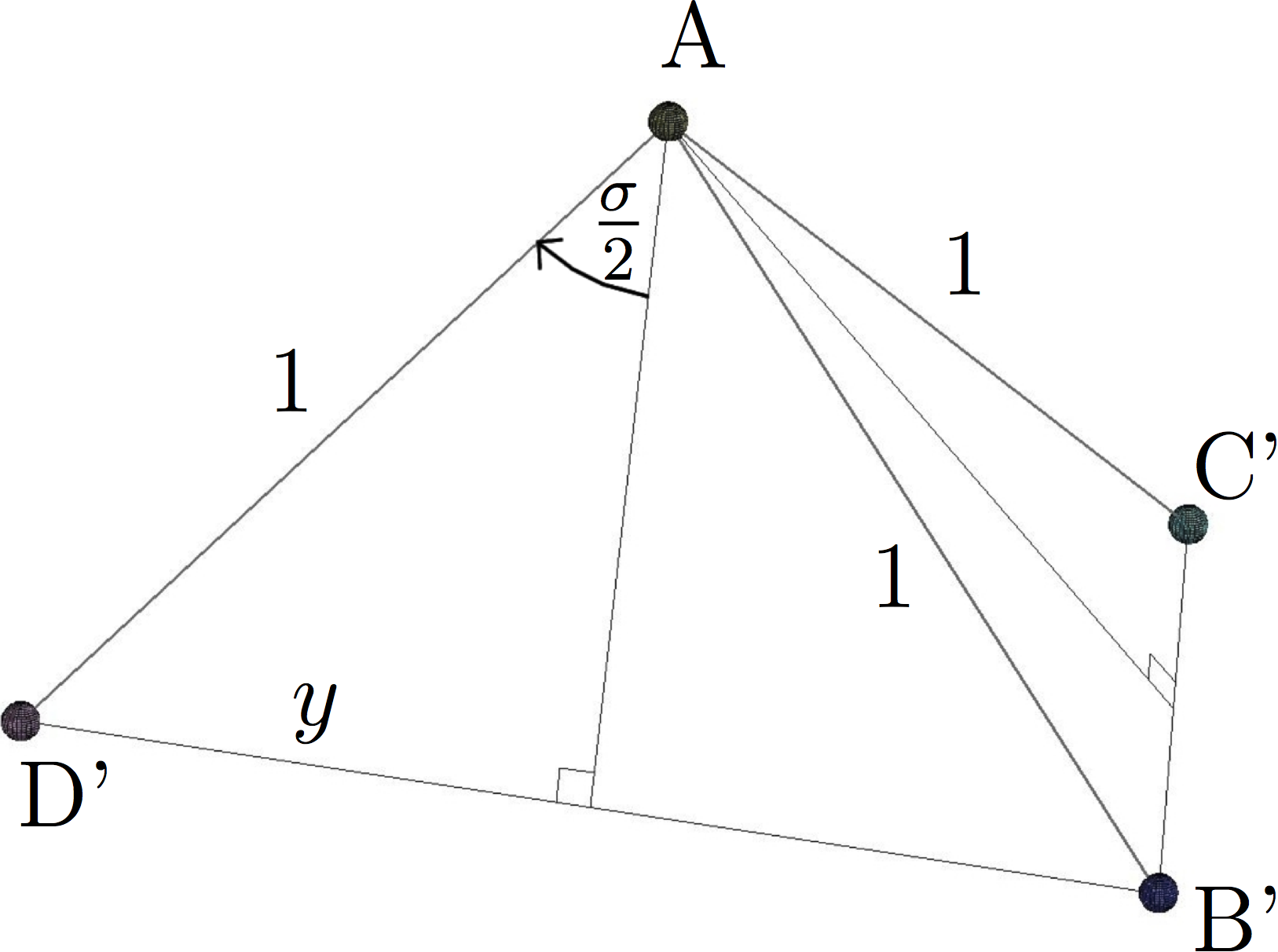

Supplement: Figure S4 — Definition of the distance . Note that is the angle DAB, , so . (TIFF) [file pcbi.1002474.s004.tif]
