# Supplementary material for: Geometric Theory Predicts Bifurcations in Minimal Wiring Cost Trees in Biology Are Flat
Source: PLoS Comput Biol. 2012 Apr 12;8(4):e1002474. doi: 10.1371/journal.pcbi.1002474 (PMC3325189; doi:10.1371/journal.pcbi.1002474)
Supplement: Table S1 — Details of the experimental data used in the paper. Details of coral data can be found in [16]. Reconstructed neurons were acquired from the database Neuromorpho.org [55]. (DOC) [file pcbi.1002474.s005.doc]

| Table S1: Detailed information on the experimental data | | | | | | |
| --- | --- | --- | --- | --- | --- | --- |
| **Coral Data** | | | | | | |
| **Type of coral** | **Number of corals** | | | **Number of bifurcations** | | |
| *Madracis Carmabi* | 9 | | | 478 | | |
| *Madracis Decactis* | 10 | | | 433 | | |
| *Madracis Formosa* | 7 | | | 410 | | |
| *Madracis Mirabilis* | 3 | | | 845 | | |
| **Neuron Data** | | | | | | |
| **Type of cell** | **Area** | **Animal** | **Number of cells** | **Number of  dendritic bifurcations** | **Number of axonal bifurcations** | **Laboratories** |
| Granule cell | Hippocampus,  dentate gyrus | Rat | 43 | 649 | 0 | Claiborne, B. (Texas, US) |
| Pyramidal cell | Cerebral cortex,  Layer 2,3 somato-sensory | Rat | 48 | 1829 | 754 | Markram, H. (Lausanne, Switzerland), Svoboda, K. (Virginia, US) |
| Pyramidal cell | Cerebral cortex,  Layer 5 prefrontal | Rat | 56 | 928 | 0 | Smith, R. F. (Virginia, US) |
| Purkinje cell | Cerebellum | Rat,  Guinea pig,  Mouse | 10 (3, 3, 4) | 4092 | 0 | Häusser, M. (London, UK), Yarom, Y. (Jerusalem, Israel), Martone, M. E. (San Diego, US) |
| Basket cell | Cerebral cortex, somatosensory | Rat | 50 | 982 | 7654 | Markram, H. (Lausanne, Switzerland) |
| Alpha motor neuron | Spinal cord | Cat | 8 | 663 | 0 | Fyffe, R. E.  (Ohio, US) |
| Martinotti cell | Cerebral cortex, somatosensory | Rat | 29 | 880 | 4931 | Markram, H. (Lausanne, Switzerland) |
| Bitufted cell | Cerebral cortex, somatosensory | Rat | 15 | 391 | 1664 | Markram, H. (Lausanne, Switzerland) |
