# Supplementary material for: Geometric Theory Predicts Bifurcations in Minimal Wiring Cost Trees in Biology Are Flat
Source: PLoS Comput Biol. 2012 Apr 12;8(4):e1002474. doi: 10.1371/journal.pcbi.1002474 (PMC3325189; doi:10.1371/journal.pcbi.1002474)
Supplement: Table S2 — Comparisons between bifurcations with different orientations with respect to Z-plane. First two columns: % of bifurcations that belonged to each group. Third column: Jensen-Shannon divergence between the cone angle distributions for the two experimental groups (* two distributions are not different from each other, Kolmogorov-Smirnov test p-value = 0.01). Last two columns: Jensen-Shannon divergence of experimental groups with the cone angle distribution for random bifurcations. (DOC) [file pcbi.1002474.s006.doc]

| Table S2: Comparison between bifurcations with different orientations with respect to Z-plane | | | | | |
| --- | --- | --- | --- | --- | --- |
|  | G1 %  (0°-30°) | G3 % (60°-90°) | JSDiv (G1, G3) | JSDiv (G1, R) | JSDiv (G3, R) |
| Granule cells | 46 % | 22 % | 0.01^*^ | 0.29 | 0.28 |
| L2/3 somatosensory pyramidal | 21 % | 41 % | 0.01^*^ | 0.19 | 0.18 |
| L5 PFC pyramidal | 65 % | 9 % | 0.04 | 0.26 | 0.17 |
| Purkinje cell | 91 % | 2 % | 0.10 | 0.28 | 0.14 |
| Basket cell | 24 % | 33 % | 0.01^*^ | 0.17 | 0.15 |
| Alpha motor neuron | 38 % | 30 % | 0.02 | 0.25 | 0.19 |
| Martinotti cell | 29 % | 32 % | 0.02^*^ | 0.20 | 0.14 |
| Bitufted cell | 28 % | 39 % | 0.01^*^ | 0.19 | 0.15 |
