# Supplementary material for: Geometric Theory Predicts Bifurcations in Minimal Wiring Cost Trees in Biology Are Flat
Source: PLoS Comput Biol. 2012 Apr 12;8(4):e1002474. doi: 10.1371/journal.pcbi.1002474 (PMC3325189; doi:10.1371/journal.pcbi.1002474)
Supplement: Text S1 — This file contains computing cone angle from the first bifurcation segment, assessing the possible effect of shrinkage artefacts in neuronal reconstruction data, extended comments relating to the proof, numerical evaluation of the cone angle and cone angle distribution for random points. (PDF) [file pcbi.1002474.s007.pdf]

Geometric theory predicts bifurcations in minimal wiring cost trees are flat

Yihwa Kim, Robert Sinclair, Nol Chindapol, Jaap A. Kaandorp & Erik De Schutter

Supporting Information (General)

Computing cone angles from the first bifurcation segments

In the main text (Figure 1) cone angles were computed for complete branches, i.e. for each branch from the bifurcation point to the next bifurcation or terminal point, because these points are morphologically well defined. In Figure S2 we show the same analysis but for bifurcations to the closest point available in the reconstruction. These first points were selected by those who performed each neuron reconstruction and the corresponding segment lengths tend to be quite variable. Though the proportion of planar bifurcations are smaller than in Figure 1, the difference with random bifurcations remains highly significant for all (KS-test, p-value = 10^{-5}).

Assessing the possible effect of shrinkage artefacts in neuronal reconstruction data

The dehydration of slices during the histological preparation can induce a pronounced shrinkage in the z-dimension, while shrinkages in other dimensions (x and y) may be much smaller [1,2]. To address the issue of shrinkage [1,2,3,4,5] as well as the systematic errors introduced by histological processes [6], both of which would influence mainly the z-coordinates, we compared bifurcations having planes with different orientations relative to the Z-plane. The bifurcation planes were categorized into 3 groups with bin centers at 15, 45 and 75°. The cone angle distributions of 2

groups, with planes close to vertical (bin center = 75°) or horizontal (bin center = 15°), were compared with each other as well as with that of random bifurcations (eq. 1, main paper). We hypothesized that if shrinkage had an effect on the shape of bifurcations then the cone angle distribution of vertically and horizontally oriented bifurcations should be different.

The results of this analysis are presented in Table S2. In all the neurons examined, the differences between the vertically and horizontally oriented experimental groups were much smaller than the differences with the random distribution. This suggests that the significantly larger proportion of planar bifurcations in the experimental data compared to random bifurcations (Figure 2C) was not caused by compression artefacts. Moreover in 5 neuron types there was no significant difference between the two experimental groups (KS test, p-value = 0.01). The neurons where the vertically and horizontally oriented experimental groups showed differences were Purkinje cells, Layer 5 PFC pyramidal cells and alpha motor neurons. In the case of Purkinje cells, since most bifurcations had the same orientation due to their planar dendritic tree this result was expected. In the case of Layer 5 PFC pyramidal cells (with a clearly underrepresented vertically oriented group) and alpha motor neurons, the data were obtained from very thin slices and we speculate that the neural reconstructions suffered from extensive cutting artefacts [7], causing a bias in the distribution.

Supporting References

1. Hellwig B (2000) A quantitative analysis of the local connectivity between pyramidal neurons in layers 2/3 of the rat visual cortex. Biol Cybern 82: 111-121.
2. Egger V, Nevian T, Bruno RM (2008) Subcolumnar dendritic and axonal organization of spiny stellate and star pyramid neurons within a barrel in rat somatosensory cortex. Cereb cortex 18: 876-889.
3. Sultan F, Czubayko U, Thier P (2002) Morphological classification of the rat lateral cerebellar nuclear neurons by principal component analysis. The Journal of comparative neurology 455: 139-155.
4. Li Y, Berewer D, Burken RE, Ascoli GA (2005) Developmental changes in spinal motoneuron dendrites in neonatal mice. The Journal of comparative neurology 483: 304-317.
5. Steuber V, De Schutter E, Jaeger D (2004) Passive models of neurons in the deep cerebellar nuclei: the effect of reconstruction errors. Neurocomputing 58-60: 563-568.
6. Jacobs G, Claiborne B, Harris K (2009) Reconstruction of neuronal morphology. In: De Schutter E, editor. Computational modeling methods for neuroscientists: MIT Press. pp. 187-210.
7. Anwar H, Riachi I, Hill S, Schuermann F, Makram H (2009) An approach to capturing neuron morphological diversity. In: De Schutter E, editor. Computational modeling methods for neuroscientists: The MIT Press. pp. 211-232.
8. Filatov MV, Kaandorp JA, Postma M, Van Liere R, Kruszynski KJ, et al. (2010) A comparison between coral colonies of the genus *Madracis* and simulated forms. Proc R Soc B 277: 3555-3561.
9. Ascoli GA, Donohue DE, Halavi M (2007) Neuromorpho.org: A central resource for neuronal morphologies. J Neurosci 27: 9247-9251.

Geometric theory predicts dendritic bifurcations in minimal wiring cost trees are flat

Yihwa Kim, Robert Sinclair, Nol Chindapol, Jaap A. Kaandorp & Erik De Schutter

Supporting Information (Mathematical)

This part of the Supporting Information consists of three self-contained parts. The first part relates to the proof of planarity of bifurcations in minimal wiring cost trees contained in the main text. The second is a discussion of numerical algorithms for computing the cone angle. The third is the calculation of the distribution of cone angles for randomly distributed points.

All parts have been written with readers familiar with mathematics in mind, but not exclusively for professional mathematicians, who may notice a lack of details and also a lack of elegant tricks. The intention throughout has been to make it possible for an interested reader to follow the mathematical line of thought without requiring any advanced expert knowledge.

5 Extended Comments relating to the Proof

5.1 Application of the proof

It has been known for some time (1) that optimal planar bifurcations, involving edges with different costs per unit length, do not exhibit 120° bifurcation angles in general. Our result provides a theoretical justification for the application of such planar algorithms to three-dimensional problems.

What our result makes clear is that it is not the fact that edges make 120° angles at bifurcation points in minimal Steiner trees which should be regarded as fundamental, but rather the fact that deformable bifurcations are always planar in minimal Steiner trees and indeed all wiring cost optimal trees.

5.2 Mechanistic explanations of bifurcation planarity

There are contexts in which mechanistic explanations of bifurcation planarity are appropriate (2). It should however be stressed that our proof does not require the existence of any construct which might be called a force (this would be some derivative of wiring cost which would always indicate the direction in which change must occur in order that cost be reduced), since we do not require that the edge cost functions $f_{i,j}$ be differentiable everywhere, nor that their derivative be positive whenever defined. The counter-intuitive function defined in (3) provides an example for which no meaningful force could be defined, but to which our method still applies. To gain some idea of what is meant by this, it is

helpful to imagine a long staircase with horizontal steps. Although one can easily understand that the bottom of the stairway is lower than the top, a tiny ball placed upon any horizontal step will not roll down. That is, the ball feels no force pulling it to the bottom. The counter-intuitive function is a limit of stairways in which initially downward sloping steps are becoming not only more in number but also more horizontal as construction proceeds. Therefore, we have shown that bifurcation planarity cannot automatically be ascribed to the action of forces.

5.3 Relation to the Euclidean Steiner tree problem

The Euclidean Steiner tree problem (4) for M given points, A_1 to A_M, and N Steiner points, S_1 to S_N, is a special case of our optimization problem [**3**], and our planarity result is already well known in this classical, restricted context (4, 5). What is new in our work is the focus on a specific type of problem from the natural sciences, which has guided our investigation towards a specific and immediately applicable approach to generalizing the Steiner tree problem. In this way, we have been able to avoid certain problems associated with over-generalization. We have also focused on a specific question in this context: Whether local bifurcation planarity could be due to a global optimization principle. We have been able to do so without needing to confront the NP-complete optimization problem which is at the heart of the Steiner tree problem (6).

Despite the apparent simplicity of the problem of finding properties of a least cost network, it must be stressed that the generalization of the Steiner tree problem to spaces which are not Euclidean, which is one of the most obvious routes to cost functions more general than just Euclidean distance, is an area of active mathematical research (7). To give an idea of the challenges to be overcome, even the concept of planarity or "flatness", which lies at the heart of what we are investigating, cannot easily be defined in more general spaces. We make use of the envelope or convex hull of three points, which is flat (a triangle) in three-dimensional Euclidean space, but it is *not known* what the envelope of three points in a general three-dimensional Riemannian manifold is, even whether it is closed (see Note 6.1.3.1 in (8)).

One can see that the Steiner tree problem is a special case of our optimization problem by identifying

$$S_i \equiv v_i \qquad [\mathbf{4}]$$

for all $i \in \{1, ...N\}$, setting

$$f_{i,j}(d(v_i, v_j)) = d(v_i, v_j)\,, \qquad [\mathbf{5}]$$

for all $i, j \in \{1, ..., N + M\}$ and defining

$$R_i = \mathbb{E}^3 \qquad [\mathbf{6}]$$

for all $i \in \{1, ..., N\}$, meaning that the Steiner points are not spatially restricted, and

$$R_{N+i} = \{A_i\} \qquad [\mathbf{7}]$$

for all $i \in \{1, ..., M\}$.

5.4 Cost defined in terms of paths to a soma

Recall that we consider only connected trees. Assume that the terminal vertex v_{N+M} is the soma. Let L_i ($i \in \{N+1, N+2, ..., N+M-1\}$) be the set of edges (represented by unordered pairs of vertices) connecting the non-soma terminal point v_i to the soma. Each L_i is a set of sets. Note that the union of all these L_i will necessarily be the full set of edges of the tree. We can define a cost which is the sum over the paths from every non-soma terminal point to the soma:

$$\begin{aligned} &W^S(v_1, v_2, ..., v_N, v_{N+1}, ..., v_{N+M}) \\ &= \sum_{k=N+1}^{N+M-1} \sum_{\{i,j\}\in L_k} f^L_{i,j}(d(v_i, v_j)) \\ &= \sum_{k=N+1}^{N+M-1} \left(\frac{1}{2} \sum_{i=1}^{N+M} \sum_{j=1}^{N+M} \chi_k(i,j)\, f^L_{i,j}(d(v_i, v_j)) \right) , \end{aligned} \qquad \textbf{[8]}$$

where the second sum defines the somewhat loose notation of the first in terms of the indicator function

$$\chi_k(i,j) = \begin{cases} 1 & (\text{if} \quad \{i,j\} \in L_k) \\ 0 & (\text{if} \quad \{i,j\} \notin L_k) \,. \end{cases} \qquad \textbf{[9]}$$

The cost **[8]** can be reduced to the same form as **[2]** by introducing new edge cost functions $f^S_{i,j}$. We can write

$$\begin{aligned} W^S(v_1, v_2, ..., v_N, v_{N+1}, ..., v_{N+M}) &= \frac{1}{2} \sum_{i=1}^{N+M} \sum_{j=1}^{N+M} \left(\sum_{k=N+1}^{N+M-1} \chi_k(i,j) \right) f^L_{i,j}(d(v_i, v_j)) \\ &= \frac{1}{2} \sum_{i=1}^{N+M} \sum_{j=1}^{N+M} C(i,j)\, f^L_{i,j}(d(v_i, v_j)) \\ &= \frac{1}{2} \sum_{i=1}^{N+M} \sum_{j=1}^{N+M} f^S_{i,j}(d(v_i, v_j)) \,, \end{aligned} \qquad \textbf{[10]}$$

where

$$C(i,j) = \sum_{k=N+1}^{N+M-1} \chi_k(i,j) \qquad \textbf{[11]}$$

and

$$f^S_{i,j}(d(v_i, v_j)) = C(i,j) \times f^L_{i,j}(d(v_i, v_j)) \,. \qquad \textbf{[12]}$$

$C(i,j)$ has a simple interpretation. It counts the number of times the edge connecting vertices v_i and v_j appears in all the paths from non-soma terminal points to the soma. $C(i,j)$ does not depend upon Euclidean edge length, meaning that it plays the role of a constant in discussing the properties of the $f^S_{i,j}$. Since $C(i,j)$ is a positive integer whenever vertices v_i and v_j are connected by an edge and otherwise zero, $C(i,j) > 0$ implies $\epsilon_{i,j} = 1$,

and $C(i,j) = 0$ implies $\epsilon_{i,j} = 0$. This allows us to insert $\epsilon_{i,j}$ into the sum [**10**] without changing its value:

$$W^S(v_1, v_2, ..., v_N, v_{N+1}, ..., v_{N+M}) = \frac{1}{2} \sum_{i=1}^{N+M} \sum_{j=1}^{N+M} \epsilon_{i,j}\, f^S_{i,j}(d(v_i, v_j))\,. \qquad [\mathbf{13}]$$

For any edge in the tree, if the $f^L_{i,j}$ are strictly increasing, then the new edge cost functions $f^S_{i,j}$ will also be strictly increasing because the corresponding $C(i,j)$ will be positive. Continuity follows because $C(i,j)$ is a constant for any given i and j. By construction (due to the symmetric definition of χ_k), $C(i,j) = C(j,i)$, meaning that, if the $f^L_{i,j}$ are symmetric with respect to their indices, then so will the new edge cost functions. Thus, the new edge cost functions $f^S_{i,j}$ satisfy all the conditions we require of edge cost functions, and we have shown that a cost defined in terms of costs of paths to the soma can be reduced to the general form [**2**] introduced in the main text.

6 Numerical Evaluation of the Cone Angle

In the following, we will sketch the arguments leading to various different formulations of the basic cone angle formula, using a notation which is reasonably close to the original description provided by Uylings and Veltman (9). See Figure S3. The point of this note is to provide mathematically equivalent but faster or numerically more stable formulations. We will make no attempt to actually carry out any numerical analysis, since that would be beyond the scope of our paper.

Uylings and Veltman (9) defined the cosine of the cone angle to be

$$\cos\alpha = \frac{4\,x^2\,y^2\,(1-2\,z^2)-(x^2+y^2-z^2)^2}{4\,x^2\,y^2-(x^2+y^2-z^2)^2}\,, \quad \textbf{[14]}$$

where

$$\begin{aligned} x &= \sin\frac{\rho}{2} \\ y &= \sin\frac{\sigma}{2} \\ z &= \sin\frac{\tau}{2} \end{aligned} \quad \textbf{[15]}$$

and the angles $\rho = \angle\mathrm{BAC}$, $\sigma = \angle\mathrm{DAB}$ and $\tau = \angle\mathrm{DAC}$ can be computed using the law of cosines:

$$\begin{aligned} \cos\rho &= \cos\angle\mathrm{BAC} = \frac{\overline{\mathrm{AB}}^2+\overline{\mathrm{AC}}^2-\overline{\mathrm{BC}}^2}{2\,\overline{\mathrm{AB}}\times\overline{\mathrm{AC}}} \\ \cos\sigma &= \cos\angle\mathrm{DAB} = \frac{\overline{\mathrm{AD}}^2+\overline{\mathrm{AB}}^2-\overline{\mathrm{DB}}^2}{2\,\overline{\mathrm{AD}}\times\overline{\mathrm{AB}}} \\ \cos\tau &= \cos\angle\mathrm{DAC} = \frac{\overline{\mathrm{AD}}^2+\overline{\mathrm{AC}}^2-\overline{\mathrm{DC}}^2}{2\,\overline{\mathrm{AD}}\times\overline{\mathrm{AC}}}\,. \end{aligned} \quad \textbf{[16]}$$

To begin with, we can simplify **[14]**:

$$\cos\alpha = 1 - \frac{8\,x^2\,y^2\,z^2}{4\,x^2\,y^2-(x^2+y^2-z^2)^2}\,. \quad \textbf{[17]}$$

6.1 An efficient formulation

Computation of the cosine of the cone angle using Equations **[14]** to **[16]** as they stand involves quite a number of sine and inverse cosine calculations. These are costly compared with more elementary arithmetic operations, and so one can ask whether they can be eliminated.

Noting that only the squares of x, y and z appear in Equations [**14**] and [**17**], we can indeed use the trigonometric identity

$$2\,\sin^2\theta = 1 - \cos 2\theta \qquad [\mathbf{18}]$$

to write

$$\begin{aligned}
\mathcal{X} = 2\,x^2 &= 2\,\sin^2\frac{\rho}{2} = 1 - \cos\rho \\
&= 1 - \frac{\overline{\mathrm{AB}}^2 + \overline{\mathrm{AC}}^2 - \overline{\mathrm{BC}}^2}{2\,\overline{\mathrm{AB}} \times \overline{\mathrm{AC}}}, \qquad [\mathbf{19}] \\
\mathcal{Y} = 2\,y^2 &= 2\,\sin^2\frac{\sigma}{2} = 1 - \cos\sigma \\
&= 1 - \frac{\overline{\mathrm{AD}}^2 + \overline{\mathrm{AB}}^2 - \overline{\mathrm{DB}}^2}{2\,\overline{\mathrm{AD}} \times \overline{\mathrm{AB}}} \qquad [\mathbf{20}] \\
\mathcal{Z} = 2\,z^2 &= 2\,\sin^2\frac{\tau}{2} = 1 - \cos\tau \\
&= 1 - \frac{\overline{\mathrm{AD}}^2 + \overline{\mathrm{AC}}^2 - \overline{\mathrm{DC}}^2}{2\,\overline{\mathrm{AD}} \times \overline{\mathrm{AC}}} \qquad [\mathbf{21}]
\end{aligned}$$

and Equation [**17**] in terms of these:

$$\cos\alpha = 1 - \frac{\mathcal{X}\mathcal{Y}\mathcal{Z}}{\mathcal{X}\mathcal{Y} - (\mathcal{X} + \mathcal{Y} - \mathcal{Z})^2/4}. \qquad [\mathbf{22}]$$

6.2 A more accurate formulation

Equations [**19**] to [**22**] do not lead to particularly accurate approximations of $\cos\alpha$. The problem we wish to address here occurs when $\mathcal{X}$, $\mathcal{Y}$ or $\mathcal{Z}$ are very small. Catastrophic cancellation (10) can result in very low accuracy.

We can significantly reduce this source of error by noting that $2\,x = \overline{B'C'}$, $2\,y = \overline{D'B'}$ (see Figure S4) and $2\,z = \overline{D'C'}$. We can then rewrite Equation [**17**] as

$$\cos\alpha = 1 - \frac{2\,s_x\,s_y\,s_z}{4\,s_x\,s_y - (s_x + s_y - s_z)^2} \qquad [\mathbf{23}]$$

where

$$\begin{aligned}
s_x &= \overline{B'C'}^2 = \left\| \frac{\vec{AC}}{\overline{AC}} - \frac{\vec{AB}}{\overline{AB}} \right\|^2 \\
s_y &= \overline{D'B'}^2 = \left\| \frac{\vec{AB}}{\overline{AB}} - \frac{\vec{AD}}{\overline{AD}} \right\|^2 \\
s_z &= \overline{D'C'}^2 = \left\| \frac{\vec{AC}}{\overline{AC}} - \frac{\vec{AD}}{\overline{AD}} \right\|^2 \qquad [\mathbf{24}]
\end{aligned}$$

and $\|\vec{v}\|$ is the Euclidean length of the vector $\vec{v}$.

In fact, we can factorize the denominator of Equation [**23**]:

$$\cos\alpha = 1 + \frac{2\, s_x\, s_y\, s_z}{\left(\sqrt{s_x}+\sqrt{s_y}+\sqrt{s_z}\right)\left(\sqrt{s_x}-\sqrt{s_y}+\sqrt{s_z}\right)\left(\sqrt{s_x}+\sqrt{s_y}-\sqrt{s_z}\right)\left(\sqrt{s_x}-\sqrt{s_y}-\sqrt{s_z}\right)}\,. \qquad [\mathbf{25}]$$

This will improve the accuracy near zeros of the denominator.

When implementing any one of the formulae presented here, it will be necessary to familiarize oneself with the numerical environment one is working in. The intention has been to provide the reader with a number of useful choices. We had had some numerical difficulties working directly with Equation [**14**].

7 Cone Angle Distribution for Random Points

It is not in general possible to define what is meant by phrases such as "cone angle distribution for points randomly distributed in space" (11). We will therefore need to begin with a clarification of what we mean in the context of our paper. We will specify a practically implementable algorithm which generates the desired distribution and show that the result is meaningful. In this algorithm, we fix the bifurcation point at the origin, and randomly and independently distribute the three other points of the bifurcation in a unit sphere centred at the origin. For each set of random points, we compute the cone angle of the bifurcation, discarding singular cases. The probability density of the cone angle, as computed by this first algorithm, will be shown to be (Equation **[1]** of the main text)

$$f(\alpha) = \frac{3}{4}\sin^3\frac{\alpha}{2}\,.$$

We define "points randomly distributed in space" in terms of a homogeneous Poisson point process (12), and consider only conditional probabilities, assuming that one point is at $(0,0,0)$. One can almost surely find a sphere centred at $(0,0,0)$ which contains exactly three other points. This sphere plays a prominent role in our calculations.

To define a cone with given vertex, we require four points in general. In the following, we assume that the origin is the cone vertex. That is, the origin is understood to be the bifurcation point. The cone angle is dependent only upon the directions from the origin to the three distinct non-bifurcation points, and the distances to the non-bifurcation points are therefore irrelevant.

Our problem is this: Given a bifurcation point fixed at the origin, and three other points distributed at random in a sphere of radius $r > 0$ centred at the origin, all four points being distinct, what is the distribution of the cone angle $0 \le \alpha \le \pi$ as defined by Uylings and Smit (13) (see Equation **[14]**)? We will provide a sketch, not a full mathematical proof. Our intention is to allow the mathematically inclined reader to convince themself that the result is in fact correct. The intuitive argument provided in the main text produces the correct answer, but avoids the issue of singular cases which arise when four points do not define a cone (the case when all four points coincide is the simplest of infinitely many cases). The point of this part of the Supporting Information is to show that these singular cases do not in fact influence the cone angle distribution.

Let the positions of the branch point, parent, and two daughters be given by

$$\vec{b} = (0,0,0) \quad \textbf{[26]}$$

$$\vec{p} = (p_x, p_y, p_z) \quad \textbf{[27]}$$

$$\vec{d_1} = (d_{1x}, d_{1y}, d_{1z}) \quad \textbf{[28]}$$

$$\text{and} \quad \vec{d_2} = (d_{2x}, d_{2y}, d_{2z})\,. \quad \textbf{[29]}$$

One can almost always find (details below) a unique cone with vertex at the origin and cone angle α which contains the three points $\vec{p}$, $\vec{d_1}$ and $\vec{d_2}$.

Let $\chi(\vec{p}, \vec{d}_1, \vec{d}_2, \alpha)$ denote the function which is equal to unity if both (i) a cone angle can be computed from $\vec{p}$, $\vec{d}_1$ and $\vec{d}_2$ and (ii) the value of this cone angle is less than or equal to α, and, otherwise, is equal to zero. We define the cumulative distribution function of the cone angle to be

$$F(\alpha) = \frac{\underset{\Lambda_r}{\int\int\int} \chi(\vec{p}, \vec{d}_1, \vec{d}_2, \alpha)\, d\vec{p}\, d\vec{d}_1\; d\vec{d}_2}{\underset{\Lambda_r}{\int\int\int} \chi(\vec{p}, \vec{d}_1, \vec{d}_2, \pi)\, d\vec{p}\, d\vec{d}_1\; d\vec{d}_2}\,, \qquad \textbf{[30]}$$

where

$$\Lambda_r = \left\{ (\vec{v}_1, \vec{v}_2, \vec{v}_3) \in \left(\mathbb{E}^3\right)^3 \;\middle|\; \|\vec{v}_1\|, \|\vec{v}_2\|, \|\vec{v}_3\| \leq r \right\} . \qquad \textbf{[31]}$$

When a unique cone is defined, we can reparametrize in terms of the cone angle α, the distances from the origin to the points $\vec{p}$, $\vec{d}_1$ and $\vec{d}_2$ (which we shall denote as $\ell_p \equiv \|\vec{p}\|$, $\ell_{d_1} \equiv \|\vec{d}_1\|$ and $\ell_{d_2} \equiv \|\vec{d}_2\|$ respectively) and the angles Ψ, Φ, θ_p, θ_{d_1} and θ_{d_2}:

$$\begin{aligned} \vec{p} &= \ell_p \times (\,\vec{c} + \cos\theta_p \times \vec{x} + \sin\theta_p \times \vec{y}\,) && \textbf{[32]} \\ \vec{d}_1 &= \ell_{d_1} \times (\,\vec{c} + \cos\theta_{d_1} \times \vec{x} + \sin\theta_{d_1} \times \vec{y}\,) && \textbf{[33]} \\ \text{and} \qquad \vec{d}_2 &= \ell_{d_2} \times (\,\vec{c} + \cos\theta_{d_2} \times \vec{x} + \sin\theta_{d_2} \times \vec{y}\,)\;, && \textbf{[34]} \end{aligned}$$

where the orthogonal set of auxiliary vectors $\vec{c}$, $\vec{x}$ and $\vec{y}$ is defined as

$$\begin{aligned} \vec{c} &= (\,\cos\Psi,\; \sin\Psi\,\cos\Phi,\; \sin\Psi\,\sin\Phi\,)\,\cos\frac{\alpha}{2} && \textbf{[35]} \\ \vec{x} &= (\,-\sin\Psi,\; \cos\Psi\,\cos\Phi,\; \cos\Psi\,\sin\Phi\,)\,\sin\frac{\alpha}{2} && \textbf{[36]} \\ \text{and} \qquad \vec{y} &= (\,0,\; -\sin\Phi,\; \cos\Phi\,)\,\sin\frac{\alpha}{2}\,. && \textbf{[37]} \end{aligned}$$

Note that $\vec{c}$ defines the axis of the cone.

Equations [**32**] to [**37**] define a smooth map

$$M : (\alpha, \Psi, \Phi, \theta_p, \theta_{d_1}, \theta_{d_2}, \ell_p, \ell_{d_1}, \ell_{d_2}) \mapsto (p_x, p_y, p_z, d_{1x}, d_{1y}, d_{1z}, d_{2x}, d_{2y}, d_{2z})\,. \qquad \textbf{[38]}$$

This map is always defined, but its inverse is not. To see this, we can formally construct

an inverse:

$$\alpha = 2\arccos\|\vec{c}\| \quad [\mathbf{39}]$$

$$\Psi = \arccos\frac{c_x}{\|\vec{c}\|} \quad [\mathbf{40}]$$

$$\Phi = \mathrm{Arg}\left(c_y + \sqrt{-1}\times c_z\right) \quad [\mathbf{41}]$$

$$\theta_p = \mathrm{Arg}\left(\left(\frac{\vec{p}}{\|\vec{p}\|} - \vec{c}\right)\cdot\vec{x} + \sqrt{-1}\times\left(\frac{\vec{p}}{\|\vec{p}\|} - \vec{c}\right)\cdot\vec{y}\right) \quad [\mathbf{42}]$$

$$\theta_{d_1} = \mathrm{Arg}\left(\left(\frac{\vec{d_1}}{\|\vec{d_1}\|} - \vec{c}\right)\cdot\vec{x} + \sqrt{-1}\times\left(\frac{\vec{d_1}}{\|\vec{d_1}\|} - \vec{c}\right)\cdot\vec{y}\right) \quad [\mathbf{43}]$$

$$\theta_{d_2} = \mathrm{Arg}\left(\left(\frac{\vec{d_2}}{\|\vec{d_2}\|} - \vec{c}\right)\cdot\vec{x} + \sqrt{-1}\times\left(\frac{\vec{d_2}}{\|\vec{d_2}\|} - \vec{c}\right)\cdot\vec{y}\right) \quad [\mathbf{44}]$$

$$\ell_p = \|\vec{p}\| \quad [\mathbf{45}]$$

$$\ell_{d_1} = \|\vec{d_1}\| \quad [\mathbf{46}]$$

$$\ell_{d_2} = \|\vec{d_2}\|, \quad [\mathbf{47}]$$

where $\mathrm{Arg}(u + \sqrt{-1}\times v)$ is the principal value of the argument of the complex number $u + \sqrt{-1}\times v$ (assuming $u, v \in \mathbb{R}$), with values in the interval $]-\pi, \pi]$, and

$$\vec{\delta_1} = \frac{\vec{d_1}}{\|\vec{d_1}\|} - \frac{\vec{p}}{\|\vec{p}\|} \quad [\mathbf{48}]$$

$$\vec{\delta_2} = \frac{\vec{d_2}}{\|\vec{d_2}\|} - \frac{\vec{p}}{\|\vec{p}\|} \quad [\mathbf{49}]$$

$$\vec{c} = (c_x, c_y, c_z) = \frac{\vec{p}\cdot\left(\vec{\delta_1}\times\vec{\delta_2}\right)}{\|\vec{p}\|\times\|\vec{\delta_1}\times\vec{\delta_2}\|^2}\,\vec{\delta_1}\times\vec{\delta_2} \quad [\mathbf{50}]$$

$$\vec{y} = \frac{(0, -c_z, c_y)}{\sqrt{c_y^2 + c_z^2}}\sin\arccos\|\vec{c}\| \quad [\mathbf{51}]$$

$$\text{and} \quad \vec{x} = \frac{\vec{y}\times\vec{c}}{\|\vec{c}\|}. \quad [\mathbf{52}]$$

When is this inverse not defined?

- Equations [**48**], [**49**] and [**50**] require that $\|\vec{d_1}\| \neq 0$, $\|\vec{d_2}\| \neq 0$ and $\|\vec{p}\| \neq 0$, giving us $\ell_{d_1} \neq 0$, $\ell_{d_2} \neq 0$ and $\ell_p \neq 0$. This is in fact already required in order for the four points $\vec{b}$, $\vec{d_1}$, $\vec{d_2}$ and $\vec{p}$ to be distinct.
- Equation [**50**] requires that $\vec{\delta_1}\times\vec{\delta_2} \neq \vec{0}$. Keeping in mind that $\vec{d_1}/\|\vec{d_1}\|$, $\vec{d_2}/\|\vec{d_2}\|$ and $\vec{p}/\|\vec{p}\|$ are all unit vectors, we find that this is equivalent to

$$\frac{\vec{d_1}}{\|\vec{d_1}\|} \neq \frac{\vec{d_2}}{\|\vec{d_2}\|} \quad\text{and}\quad \frac{\vec{d_1}}{\|\vec{d_1}\|} \neq \frac{\vec{p}}{\|\vec{p}\|} \quad\text{and}\quad \frac{\vec{d_2}}{\|\vec{d_2}\|} \neq \frac{\vec{p}}{\|\vec{p}\|}, \quad [\mathbf{53}]$$

which translates into (see Equations [**32**] to [**34**]) $\theta_{d_1} \neq \theta_{d_2}$, $\theta_{d_1} \neq \theta_p$, $\theta_{d_2} \neq \theta_p$ and $(\vec{x}, \vec{y}) \neq (\vec{0}, \vec{0})$ (also required by Equations [**42**] to [**44**]), or, equivalently, $\alpha \neq 0$, as one can see by studying equations [**36**] and [**37**].

- Equations [**40**] and [**52**] require that $\|\vec{c}\| \neq 0$. Equation [**35**] then implies that $\alpha \neq \pi$, while Equation [**50**] can only guarantee $\|\vec{c}\| \neq 0$ if

$$\vec{p} \cdot \left(\left(\frac{\vec{d_1}}{\|\vec{d_1}\|} - \frac{\vec{p}}{\|\vec{p}\|} \right) \times \left(\frac{\vec{d_2}}{\|\vec{d_2}\|} - \frac{\vec{p}}{\|\vec{p}\|} \right) \right) \neq 0 \,, \qquad [\mathbf{54}]$$

which is equivalent to

$$\vec{p} \cdot \left(\vec{d_1} \times \vec{d_2} \right) \neq 0 \,. \qquad [\mathbf{55}]$$

- Equation [**51**] requires more than just $\|\vec{c}\| \neq 0$. It also requires that $(c_y, c_z) \neq (0,0)$, or

$$\begin{pmatrix} (0,1,0) \cdot \left\{ \left(\frac{\vec{d_1}}{\|\vec{d_1}\|} - \frac{\vec{p}}{\|\vec{p}\|} \right) \times \left(\frac{\vec{d_2}}{\|\vec{d_2}\|} - \frac{\vec{p}}{\|\vec{p}\|} \right) \right\} , \\ (0,0,1) \cdot \left\{ \left(\frac{\vec{d_1}}{\|\vec{d_1}\|} - \frac{\vec{p}}{\|\vec{p}\|} \right) \times \left(\frac{\vec{d_2}}{\|\vec{d_2}\|} - \frac{\vec{p}}{\|\vec{p}\|} \right) \right\} \end{pmatrix} \neq (0,0) \,. \qquad [\mathbf{56}]$$

This is equivalent to $\Psi \neq 0, \pi$.

These inequalities define the singular cases which must be avoided. By studying each one in isolation, the reader can convince themself that none of them define a region to be avoided which is more than what one might loosely call a slice of zero width through the domain of integration of [**30**]. What this means is that the cases in which the inverse is not defined are of zero measure with respect to the integral [**30**] – the cumulative distribution function of the cone angle – and therefore make no difference to the values of $F(\alpha)$.

The absolute value of the Jacobian determinant associated with our reparametrisation (Equations [**32**] to [**37**]) is given by

$$\sin^3\left(\frac{\alpha}{2}\right) \times \frac{J}{2} \,, \qquad [\mathbf{57}]$$

where

$$J = |\sin\Psi \times (\sin(\theta_{d_1} - \theta_p) + \sin(\theta_p - \theta_{d_2}) + \sin(\theta_{d_2} - \theta_{d_1}))| \times \ell_p^2 \, \ell_{d_1}^2 \, \ell_{d_2}^2 \qquad [\mathbf{58}]$$

(the result of a long but tedious calculation, involving the determinant of a 9×9 matrix).

The cumulative distribution function of the cone angle is given by an integral which excludes the cases (of zero measure) listed above. In an abuse of notation, we write

$$
\begin{aligned}
F(\alpha) &= \frac{\int_0^r\int_0^r\int_0^r\int_0^{2\pi}\int_0^{2\pi}\int_0^{2\pi}\int_0^{2\pi}\int_0^{\pi}\int_0^{\alpha} \sin^3\frac{a}{2}\, J\, \mathrm{d}a\, \mathrm{d}\Psi\, \mathrm{d}\Phi\, \mathrm{d}\theta_p \mathrm{d}\theta_{d_1} \mathrm{d}\theta_{d_2} \mathrm{d}\ell_p \mathrm{d}\ell_{d_1} \mathrm{d}\ell_{d_2}}{\int_0^r\int_0^r\int_0^r\int_0^{2\pi}\int_0^{2\pi}\int_0^{2\pi}\int_0^{2\pi}\int_0^{\pi}\int_0^{\pi} \sin^3\frac{a}{2}\, J\, \mathrm{d}a\, \mathrm{d}\Psi\, \mathrm{d}\Phi\, \mathrm{d}\theta_p \mathrm{d}\theta_{d_1} \mathrm{d}\theta_{d_2} \mathrm{d}\ell_p \mathrm{d}\ell_{d_1} \mathrm{d}\ell_{d_2}} \\
&= \frac{\int_0^\alpha \sin^3\left(\frac{a}{2}\right)\, \mathrm{d}a}{\int_0^\pi \sin^3\left(\frac{a}{2}\right)\, \mathrm{d}a} = \frac{3}{4}\int_0^\alpha \sin^3\left(\frac{a}{2}\right)\, \mathrm{d}a\,, \qquad \textbf{[59]}
\end{aligned}
$$

with the understanding that the singular cases have been excluded. Note that the result is independent of r, as expected.

We may now finally conclude that the probability density of the cone angle α is (this is Equation [**1**] of the main text)

$$f(\alpha) = \frac{3}{4}\sin^3\frac{\alpha}{2}\,.$$

7.1 Points distributed on the surface of a sphere

If we fix $\|\vec{d_1}\| = \|\vec{d_2}\| = \|\vec{p}\| = r$ (where, as before $r > 0$), then the appropriate cumulative distribution function is

$$
\begin{aligned}
F_{\text{surface}}(\alpha) &= \frac{\int_0^{2\pi}\int_0^{2\pi}\int_0^{2\pi}\int_0^{2\pi}\int_0^{\pi}\int_0^{\alpha} \sin^3\frac{a}{2}\, J_{\text{surface}}\, \mathrm{d}a\, \mathrm{d}\Psi\, \mathrm{d}\Phi\, \mathrm{d}\theta_p \mathrm{d}\theta_{d_1} \mathrm{d}\theta_{d_2}}{\int_0^{2\pi}\int_0^{2\pi}\int_0^{2\pi}\int_0^{2\pi}\int_0^{\pi}\int_0^{\pi} \sin^3\frac{a}{2}\, J_{\text{surface}}\, \mathrm{d}a\, \mathrm{d}\Psi\, \mathrm{d}\Phi\, \mathrm{d}\theta_p \mathrm{d}\theta_{d_1} \mathrm{d}\theta_{d_2}} \\
&= \frac{\int_0^\alpha \sin^3\left(\frac{a}{2}\right)\, \mathrm{d}a}{\int_0^\pi \sin^3\left(\frac{a}{2}\right)\, \mathrm{d}a} = \frac{3}{4}\int_0^\alpha \sin^3\left(\frac{a}{2}\right)\, \mathrm{d}a\,, \qquad \textbf{[60]}
\end{aligned}
$$

where we once again understand that the singular cases have been excluded, and

$$J_{\text{surface}} = |\sin\Psi \times (\,\sin(\theta_{d_1} - \theta_p) + \sin(\theta_p - \theta_{d_2}) + \sin(\theta_{d_2} - \theta_{d_1})\,)| \times r^6\,, \qquad \textbf{[61]}$$

meaning that $F_{\text{surface}}(\alpha)$ does not depend upon the value of r. Thus, the cone angle distribution is the same as Equation [**1**] of the main text.

Supporting Information (Mathematical) References

(1) Murray C (1926) The Physiological Principle of Minimum Work applied to the Angle of Branching of Arteries. *J Gen Psychol* 9:835–841.

(2) Yin Y, Chen Y, Yin J, Huang K (2006) Geometric conservation laws for perfect Y-branched carbon nanotubes. *Nanotechnology* 17:4941–4945.

(3) Salem R (1943) On Some Singular Monotonic Functions Which Are Strictly Increasing. *Trans Am Math Soc* 53:427–439.

(4) Gilbert E, Pollak H (1968) Steiner Minimal Trees. *SIAM J Appl Math* 16:1–29.

(5) Toppur B, MacGregor Smith J (2005) A Sausage Heuristic for Steiner Minimal Trees in Three-Dimensional Euclidean Space. *J Math Model Algorithm* 4:199–217.

(6) Karp R (1972) in Complexity of Computer Computations, eds Miller R, Thatcher J (Plenum Press, New York), pp 85–103.

(7) Innami N, Kim B (2006) Steiner ratio for hyperbolic surfaces. *Proc Japan Acad Ser A Math Sci* 82:77–79.

(8) Berger M (2003) A Panoramic View of Riemannian Geometry (Springer-Verlag, Berlin).

(9) Uylings H, Veltman W (1975) Characterizing a dendritic bifurcation. *Neurosci Lett* 1:128–130.

(10) Goldberg D (1991) What Every Computer Scientist Should Know About Floating-Point Arithmetic. *ACM Comput Surv* 23:5–48.

(11) Pfiefer R (1989) The Historical Development of J. J. Sylvester's Four Point Problem. *Mathematics Magazine* 62:309–317.

(12) Illian J, Penttinen A, Stoyan H, Stoyan D (2008) *Statistical Analysis and Modelling of Spatial Point Patterns* (Wiley, Chichester, UK).

(13) Uylings H, Smit G (1975) Three-dimensional branching structure of pyramidal cell dendrites. *Brain Res* 87:55–60.
